# A Novel POSS-Based Copolymer Functionalized Graphene: An Effective Flame Retardant for Reducing the Flammability of Epoxy Resin

**DOI:** 10.3390/polym11020241

**Published:** 2019-02-01

**Authors:** Min Li, Hong Zhang, Wenqian Wu, Meng Li, Yiting Xu, Guorong Chen, Lizong Dai

**Affiliations:** Fujian Provincial Key Laboratory of Fire Retardant Materials, College of Materials, Xiamen University, Xiamen, Fujian 361005, China; 20720161150023@stu.xmu.edu.cn (M.L.); 20720171150104@stu.xmu.edu.cn (H.Z.); 20720161150026@stu.xmu.edu.cn (W.W.); 20720171150098@stu.xmu.edu.cn (M.L.); grchen@xmu.edu.cn (G.C.)

**Keywords:** POSS-based copolymer, graphene oxide, flame retardant, epoxy resin

## Abstract

In this study, a novel copolymer, PbisDOPOMA-POSSMA-GMA (PDPG), containing methacryloisobutyl polyhedral oligomeric silsesquioxane (POSSMA), reactive glycidyl methacrylate (GMA), and bis-9,10-dihydro-9-oxa-10-phosphaphenanthrene-10-oxide methacrylate (bisDOPOMA) and derivative functionalized graphene oxide (GO) were synthesized by a one-step grafting reaction to create a hybrid flame retardant (GO-MD-MP). GO-MD-MP was characterized by Fourier transform infrared spectroscopy (FT-IR), X-ray diffraction (XRD), transmission electron microscopy (TEM), Raman spectroscopy, X-ray photoelectron spectroscopy (XPS), and thermogravimetric analysis (TGA). Flame-retardant epoxy resin (EP) composites were prepared by adding various amounts of GO-MD-MP to the thermal-curing epoxy resin of diglycidyl ether of bisphenol A (DGEBA, trade name E-51). The thermal properties of the EP composites were remarkably enhanced by adding the GO-MD-MP, and the residue char of the epoxy resin also increased greatly. With the incorporation of 4 wt % GO-MD-MP, the limiting oxygen index (LOI) value was enhanced to 31.1% and the UL-94 V-0 rating was easily achieved. In addition, the mechanical strength of the epoxy resin was also improved.

## 1. Introduction

Epoxy resins (EP) are widely used in industrial applications, such as surface coatings, castable materials, injection molding materials, and microelectronics packaging materials, due to their excellent mechanical, adhesive, and electrical insulation properties [1,2,3]. However, the fire hazards of an EP composite significantly limit its applications [4,5]. In order to reduce the risk of fire, many flame retardants have been developed, including halogen flame retardants, halogen-free flame retardants, and organic–inorganic hybrid flame retardants [6,7,8]. Halogen atoms, especially bromine or chlorine, reduce the exothermic combustion process by blocking gas-phase free-radical chemistry and inhibit combustion in the vapor phase [9]. Unfortunately, the toxicity of the hydrogen halide and brominated furans and dioxins formed during combustion were seriously harmful to human health [10]. Therefore, halogen-free flame retardants have been developed for avoiding toxic gas generation and simultaneously reducing the flammability of EP composites [2].

Over the past few years, 9,10-dihydro-9-oxa-10-phosphaphenanthrene-10-oxide (DOPO) and its derivatives have attracted great attention due to their high reactivity and flame retardancy [11,12]. Technical literature extensively reports that DOPO not only enhances the flame retardancy of epoxy resin but also its thermal stability [13,14,15]. In addition, the modification of DOPO with polyhedral oligomeric silsesquioxane (POSS), which possesses a cage-like organic–inorganic hybrid structure with a 1–3 nm dimension, can further enhance its flame retardancy [16,17]. During the combustion process, POSS is embedded as a molecular enhancer in the EP matrix, retarding the combustion of the material and reducing the heat release by retarding and restricting the movement of the EP chain. Furthermore, the rich silicon oxide structure in POSS can promote the epoxy resin to form a continuous and anti-oxidation char to reduce its flammability [18,19,20].

Graphene plays an important role in terms of enhancing the mechanical properties and flame retardancy of EP composites [21,22,23]. At the same time, the effective dispersion of graphene flakes can achieve high aspect ratios in EP composites, which improves the barrier properties of EP composites during thermal degradation due to the special platelet morphology of graphene flakes [24]. Simultaneously, graphene can promote a dense and uniform carbon layer to form in the condensed phase during the combustion of the EP composites [25]. Our previous work has shown that modified graphene oxide (GO) can be better dispersed in EP composites due to the excellent interface interaction between the modified graphene oxide and the EP composites. As a nanoplate filler capable of being well-dispersed in EP, the modified GO can also be viewed structurally to improve the mechanical properties of the EP composites [26]. Chen et al. [27] synthesized a graphene-based hybrid flame retardant by surface grafting 10-dihydro-9-oxa-10-phosphaphenanthrene-10-oxide-g-(2,3-epoxypropoxy) propyltrimethoxysilane (DPP). This type of flame retardant in EP composites achieved the UL-94 V-0 rating, indicating that it is an effective flame-retardant additive for epoxy resin laminate composites. Hu et al. [28] synthesized a functionalized graphene (PD-rGO) via an in situ reaction. It was incorporated into EP to fabricate flame-retardant EP nanocomposites, and the PHRR and THR values decreased significantly by 43.0% and 30.2%, respectively.

In order to improve the flame retardancy of graphene in EP composites, many studies have reported a different functional graphene flame retardant [29,30,31,32]. However, a POSS-based copolymer modified graphene oxide hybrid flame retardant was rarely reported. In this research, a novel POSS-based polymer modified graphene oxide hybrid flame retardant, containing long-chain POSS, DOPO, and reactive glycidyl methacrylate (GMA), was synthesized. To accomplish this, first, a copolymer, PDPG, containing POSS and DOPO, was fabricated via copolymerization using POSSMA, bisDOPOMA, and GMA monomers, then PDPG was grafted to GO to obtain GO-MD-MP. The GO-MD-MP was then used as a reactive functional filler and incorporated in the EP matrix to explore its effect on the comprehensive properties of the EP composites, including flame retardancy, thermal properties, and mechanical strengths.

## 2. Experiment

### 2.1. Materials

The methacryloisobutyl POSS (POSSMA) (molecular weight = 943.64 g/mol) was purchased from the Hybrid Plastics Company (Sinoreagent Co., Shanghai, China) and was used as received. The 9,10-Dihydro-oxa-10-phosphaphenanthrene-10-oxide (DOPO) was purchased from Shanghai Eutec Chemical, Shanghai, China. The polyoxymethylene (POM), methacryloyl chloride, 4-aminophenol, 4,4′-diaminodiphenylmethane (DDM), and glycidyl methacrylate (GMA) were purchased from Sinopharm Chemical Reagent Co. Ltd., Shanghai, China. The diglycidyl ether of bisphenol A (DGEBA, trade name E-51), a kind of liquid epoxy resin, was supplied by the Wuxi Resin Factory (Wuxi, China). The triethylamine (TEA) was distilled off before use. The reagents used were of an analytical grade, unless otherwise noted.

### 2.2. Synthesis of OH-bisDOPO

**Scheme 1:**
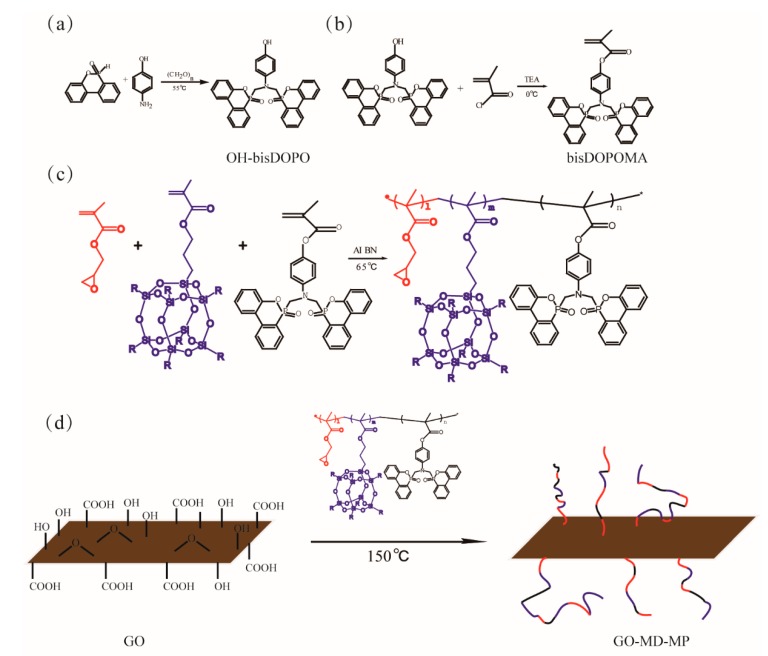
Synthetic route of OH-bisDOPO (**a**), bisDOPOMA (**b**), PDPG (**c**), and GO-MD-MP (**d**).

A volume of 200 mL of ethanol solution of DOPO (6.48 g, 30 mmol), POM (0.9 g, 30 mmol), and 4-aminophenol (1.64 g, 15 mmol) were charged into a 500 mL three-necked glass flask equipped with refluxing under argon protection. After stirring for 30 min at room temperature, the mixture was continuously stirred at 55 °C for 24 h to form a white suspension. The white suspension was then filtered with a Buchner funnel and washed three times with ethanol to obtain an off-white solid. Subsequently, the product, OH-bisDOPO, was dried in a 50 °C vacuum drying oven to give a yield of 78%. The synthetic route is shown in Scheme 1a.

**Figure 1:**
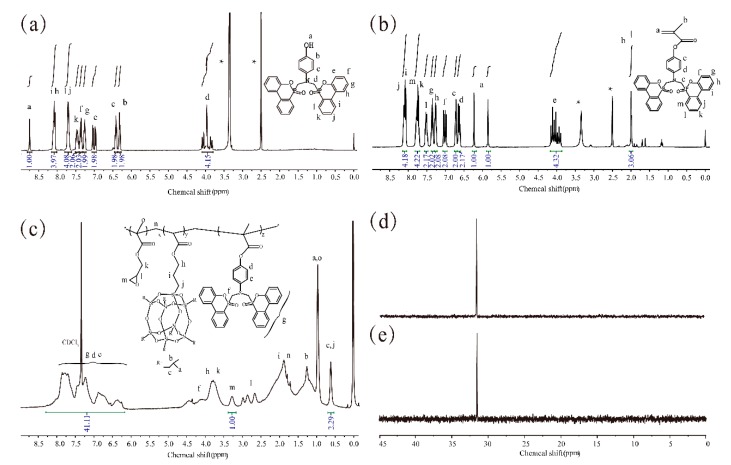
The ^1^H NMR,^31^P NMR of OH-bisDOPO (**a,d**), bisDOPOMA (**b,e**), and PDPG (**c**).

The NMR spectra of OH-bisDOPO are shown in Figure 1a: ^1^H NMR(DMSO-d6, 400 MHz) δ (ppm)—8.74 (s, 1H), 8.04~8.14 (m, 4H), 7.64~7.75 (m, 4H), 7.48 (m, 2H), 7.35 (m, 2H), 7.26 (m, 2H), 6.98 (m, 2H), 6.41 (m, 2H), 6.32 (m, 2H), and 3.97 (m, 4H); ^31^P NMR(DMSO-d6, 121MHz) δ (ppm)—31.52 (DOPO).

### 2.3. Synthesis of bisDOPOMA Monomer

OH-bisDOPO (5.7 g, 10 mmol), TEA (1.03 g, 10 mmol) were dissolved in dichloromethane (100 mL) in a 250 mL round-bottomed glass flask. The mixture was then stirred at 0 °C for 10 min and methacryloyl chloride (1.01 g, 10 mmol) was added dropwise slowly into the mixture. Subsequently, the mixture was stirred continuously at 0 °C for 1 h and then at room temperature for 24 h. After that, the solvent was washed 3 times with deionized water and dried with anhydrous magnesium sulfate. Then, the solvent was added dropwise into 300mL of hexane to obtain a faint yellow precipitate. The bisDOPOMA powders were isolated by filtration followed by drying in a vacuum at 50 °C for 24 h. The synthetic scheme of bisDOPOMA is shown in Scheme 1b.

The NMR spectra of bisDOPOMA are shown in Figure 1b: ^1^H NMR(DMSO-d6, 400MHz) δ (ppm)—8.03~8.15 (m, 4H), 7.67~7.84 (m, 4H), 7.52 (m, 2H), 7.35 (m, 2H), 7.25 (m, 2H), 7.00 (m, 2H), 6.71 (m, 2H), 6.63 (m, 2H), 6.23 (m, 1H), 5.85 (m, 1H), and 4.03 (m, 4H); ^31^P NMR (DMSO-d6, 121MHz) δ (ppm)—31.52 (DOPO).

### 2.4. Synthesis of PDPG

PDPG was synthesized with monomers of bisDOPOMA, POSSMA, and GMA via random free-radical polymerization, as shown in Scheme 1c. GMA (0.57 g, 4 mmol), POSSMA (3.8 g, 4 mmol), bisDOPOMA (10.5 g, 16 mmol), and azodiisobutyronitrile (AIBN) (0.03 g, 0.18 mmol) were added into a 250 mL three-necked glass flask equipped with refluxing under argon with 100 mL of tetrachloroethane, and the three-necked glass flask was placed in an oil bath at 65 °C for 24 h. The product was then precipitated in excess hexane and washed three times with hexane. The resulting white powder of PDPG was dried overnight in a vacuum at 50 °C for 24 h.

The NMR spectra of PDPG are shown in Figure 1c, which give the characteristic peaks of PDPG.

### 2.5. Synthesis of GO-MD-MP

GO was synthesized from flake graphite using the modified Hummers method. First, flake graphite (3 g) was added into concentrated H_2_SO_4_/H_3_PO_4_ (360:40 mL) in a 500ml three-necked glass flask. Then, the three-necked glass flask was placed in an ice bath at 0 °C. KMnO_4_ (18 g) was added slowly in order to prevent the reaction temperature from exceeding 20 °C and the dispersions were heated to 50 °C and stirred for 12 h. Afterwards, distilled water (400 mL) and 30% hydrogen peroxide (6 mL) were added slowly until the mixed solution turned bright yellow. The mixed solution was then centrifuged, and the obtained solid was washed with deionized water and centrifuged until the pH was neutral. Next, the product, GO, was freeze-dried in a vacuum freeze dryer to obtain dry GO. After that, GO (500 mg) and PDPG (2.5 g) were dissolved with dimethylformamide (DMF) (100 mL) in a 250 mL three-necked glass flask equipped with a condenser and magnetic stirrer under an argon atmosphere. The reaction solution was then heated to 150 °C for 24 h. The obtained GO-MD-MP solution was filtered and then washed with DMF, ethanol, and deionized water three times, respectively. Finally, the product was freeze-dried in a vacuum freeze dryer. The synthetic scheme of GO-MD-MP is shown in Scheme 1d.

### 2.6. Preparation of the Flame-Retardant EP Composites

We prepared the flame-retardant EP composites with 2 wt % GO-MD-MP. First, GO-MD-MP (0.5 g) was dispersed in 20 mL of chloroform and sonicated for 30 min in an ultrasonic bath. After that, epoxy resins (13.3 g) and the abovementioned GO-MD-MP dispersion in chloroform were mixed and then sonicated for 1 h at room temperature. Subsequently, the chloroform was removed by vacuum evaporation, and then DDM (2.9 g) was added into the mixture at 85 °C in a 50 mL round-bottomed flask equipped with a mechanical stirrer. Finally, the mixture was cast in an aluminum mold, when the mixture became homogeneous. The aluminum mold was then placed using a three-step curing procedure to obtain the thermosetting resins. All the samples were cured at 120 °C for 4 h and then at 140 °C for 2 h. They were then further post-cured at 180 °C for 2 h. Finally, all the samples were cooled naturally to room temperature to prevent stress cracking.

### 2.7. Characterizations

The nuclear magnetic resonance (^1^H NMR, ^31^P NMR) spectra were recorded on a Bruker Advanced II AV400 MHz NMR spectrometer (Bruker, Geneva, Switzerland) with CDCl_3_ or DMSO-d6 as the solvent. The attenuated total reflectance Fourier transform infrared (ATR-FTIR) spectra were obtained on a Nicolet Avatar 360 spectrophotometer (Thermo Fisher Scientific, Shanghai, China). The X-ray photoelectron spectroscopy (XPS) was conducted using a K-Alpha (Physical Electronics, Inc., Chanhassen, MN, USA) equipped with an Al Kα radiation source. The molecular weight of the PDPG was estimated by gel permeation chromatography (GPC) with a refractive index detector model. Tetrahydrofuran (THF) was used as an eluent at a flow rate of 1 ml/min.

The scanning electron microscopy (SEM) images were obtained using an SU-70 microscope (HITACHI, Tokyo, Japan). The fracture surfaces of the cured EP composites and the char residues of the specimens from the LOI tests were observed. The morphology and structure of the GO and GO-MD-MP were studied by transmission electron microscopy (TEM) using a JEM2100 microscope (JEOL, Tokyo, Japan).

The Raman spectroscopy measurement was performed using a HORIBA Xplora Raman spectrometer (Horiba, Lille, France) with excitation by a 532 nm laser line. The X-ray diffraction (XRD) was performed using a Panalytical X’Pert diffractometer with filtered Cu radiation. Diffractograms (Rigaku, Tokyo, Japan) were obtained in a range from 5° to 55° (2θ) at room temperature.

The thermogravimetric analysis (TGA) was performed with a Netzsch STA 409EP (Netzsch, Selb, Germany) by heating from room temperature to 800 °C at a heating rate of 10 °C/min under a nitrogen atmosphere. The differential scanning calorimeter (DSC) was performed with a Netzsch STA 449C (Netzsch, Selb, Germany) by heating from room temperature to 220 °C at a heating rate of 10 °C·min^−1^ under a nitrogen atmosphere.

The limiting oxygen index (LOI) was measured with a JF-3 instrument (Jiangning, China), and the sample bar dimensions were 100 × 6 × 4 mm^3^. The vertical burning tests were conducted with an instrument from Fire Testing Technology (Jiangning, China), England, based on the UL-94 standard, and the sample bar dimensions were 125 × 12.5 × 4 mm^3^.

The three-point bending experiment was performed on an electronic universal testing machine (AGS-X, Shimadzu, Tokyo, Japan) with a sample size of 120 × 10 × 4 mm^3^ and a crosshead speed of 2 mm/min.

## 3. Results and Discussion

### 3.1. Structural and Morphological Characterization of GO-MD-MP and Cured EP Composites

**Figure 2:**
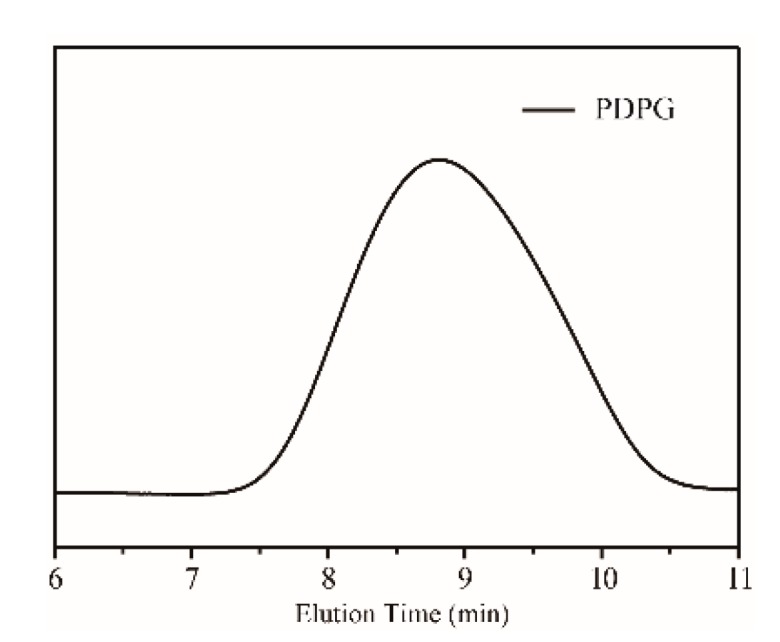
The gel permeation chromatography (GPC) trace recorded for PDPG.

The ^1^H NMR, ^31^P NMR spectra of OH-bisDOPO, bisDOPOMA, and PDPG are displayed in Figure 1. All protons can be assigned to the predicted signals. Compared to Figure 1a of OH-bisDOPO, the signal of the phenolic hydroxyl group at 8.74 ppm disappeared in Figure 1b of bisDOPOMA, and new signals at 6.23 and 5.87 ppm for bisDOPOMA were assigned to methylene protons (1H). In addition, the ^1^H NMR spectra of PDPG is shown in Figure 1c. The related characteristic peaks of PGMA, POSSMA, and bisDOPOPMA were the signals (m) at 3.23 ppm, the signals (g, d, and e) at 0.59 ppm, and the signals (c and j) at 6.2~8.2 ppm. The ratio of GMA, POSSMA, and bisDOPOMA was found to be about 1:1.15:4.11 based on the relative integration of these three kinds of signals above, revealing that the ratio of P and Si was about 0.92. The composition and structure information of PDPG is shown in Table 1. For the ^31^P NMR spectra, the signal of OH-bisDOPO and bisDOPOMA was in the same position at 31.52 ppm, which indicated that the phosphorus had one chemical environment. Figure 2 shows the GPC of PDPG. The Mn and Mw/Mn of PDPG was 16,018 and 1.55 g·mol^−1^, respectively.

**Figure 3:**
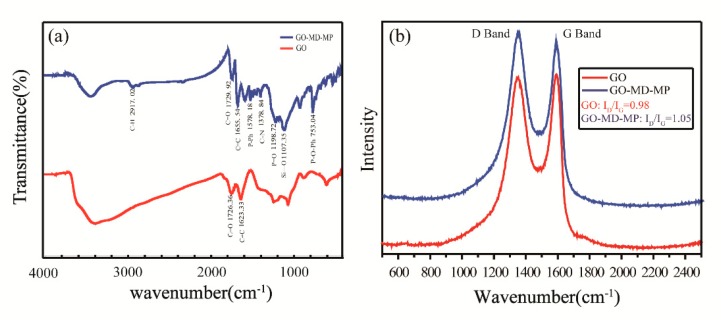
FT-IR (**a**) and Raman spectra (**b**) of graphene oxide (GO) and GO-MD-MP.

The FTIR spectra of GO and GO-MD-MP are shown in Figure 3a. The characteristic absorption peaks of GO were observed at 3400 cm^−1^ (O–H stretching vibration), 1623.33 cm^−1^(C=C stretching vibration), 1726.36 cm^−1^ (C=O stretching vibration), 1223.85 cm^−1^ (C–O of epoxy stretching vibration), and 1051.83 cm^−1^ (C–O stretching vibration of alkoxy). For GO-MD-MP, some new bands were observed at 1198.72 cm^−1^ (P=O stretching vibration), 1107.35 cm^−1^ (Si–O stretching vibration), 1378.84 cm^−1^ (C–N stretching vibration), and the typical absorption peaks between 1431 and 1600 cm^−1^ were assigned to the benzene ring. The appearance of these characteristic peaks indicated that PDPG was successfully grafted onto the GO surface.

Figure 3b shows the Raman spectroscopies of GO and GO-MD-MP. The absorption peaks appearing at 1593 and 1358 cm^−1^ correspond to the G and D bands [33], respectively. The G band represents sp^2^ hybrid carbon atoms, while the D band arises from the vibrations of the sp^3^ hybrid carbon atoms provided with the defect information for graphene [34]. The ratio of the intensities of the D and G bands (I_D_/I_G_) is a significant parameter to evaluate the structure of graphene. The I_D_/I_G_ ratio increased from 0.98 for GO to 1.05 for GO-MD-MP, indicating an increase in disordered carbon, compared to the ordered carbon structure, that was due to the PDPG graft.

**Figure 4:**
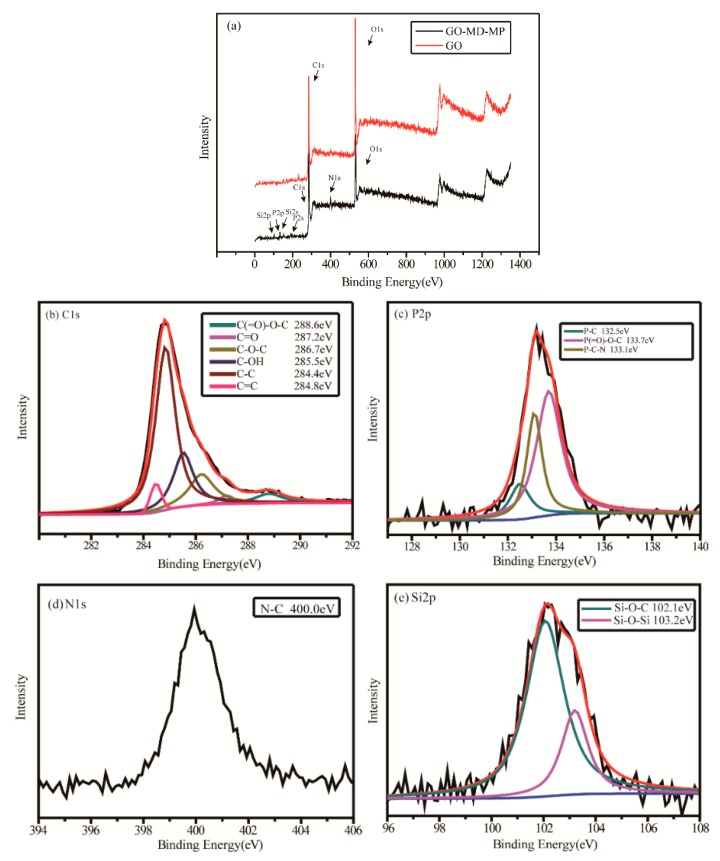
X-ray photoelectron spectroscopy (XPS) survey spectra of GO-MD-MP (**a**), C1s spectrum of GO-MD-MP (**b**), P2p spectrum of GO-MD-MP (**c**), N1s spectrum of GO-MD-MP (**d**), and Si2p spectrum of GO-MD-MP (**e**).

In order to confirm covalent functionalization, GO and GO-MD-MP were analyzed using XPS. As shown in Figure 4a, two elements, C and O, were detected in the GO spectrum. However, five elements, C, O, P, N, and Si, were observed in GO-MD-MP. Moreover, the C1s, N1s, P2p, and Si2p spectra are elucidated in Figure 4b–e, respectively. In the high-resolution C1s spectrum of GO-MD-MP, six absorbance peaks were distinguished: 284.4 and 284.8 eV were assigned to C=C and C–C in the GO skeleton, 285.5 and 286.7 eV were attributed to C–OH and C–O–C, respectively, 287.2 eV was attributed to C=O, and 288.6 eV was assigned to the C(=O)–O–C band. In the spectrum of N1s, only one peak (N–C) at 400.4 eV was detected. Moreover, in the spectrum of P2p, three peaks at 132.5, 133.7 eV, and 133.1 eV were assigned to P–C, P(=O)–O–C, and P–C–N, respectively. For the Si2p spectrum, two peaks at 102.1 and 103.2 eV were attributed to Si–O–C and Si–O–Si, respectively. In addition, the atom percentages of the various elements in GO-MD-MP were 75.32 at% (C), 20.57 at% (O), 1.01 at% (N), 1.48 at% (P), and 1.62 at% (Si), respectively. The ratio of P and Si was 0.91, which approximately coincided with the value calculated by ^1^H NMR, indicating that the graft reaction was successful. Thus, according to the measured P (1.48 at%) and Si (1.62 at%) content, the calculated grafting ratio of bisDOPOMA and POSSMA on GO was around 35.38 wt % and 14.41 wt %, respectively.

**Figure 5:**
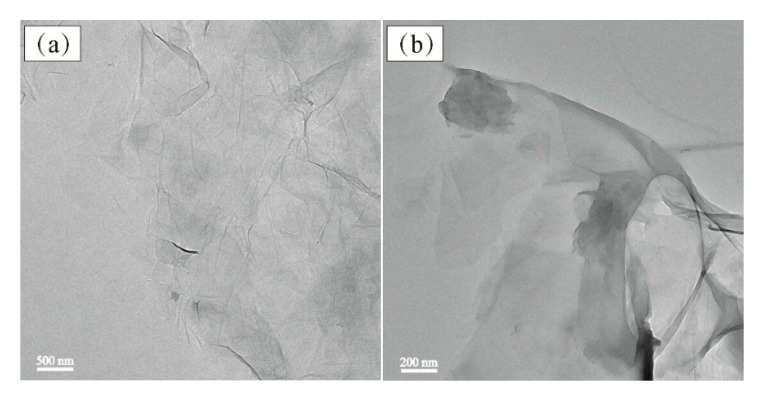
TEM images of GO (**a**) and GO-MD-MP (**b**).

TEM analysis was carried out to study the morphology of the synthesized GO-MD-MP and GO, as shown in Figure 5. GO exhibited folded sheet-like structures, as shown in Figure 5a. Moreover, Figure 5b represents the TEM image of GO-MD-MP where the dark-colored agglomerates are the modified PDPG and the light-colored layers are the graphene sheet. 

**Figure 6:**
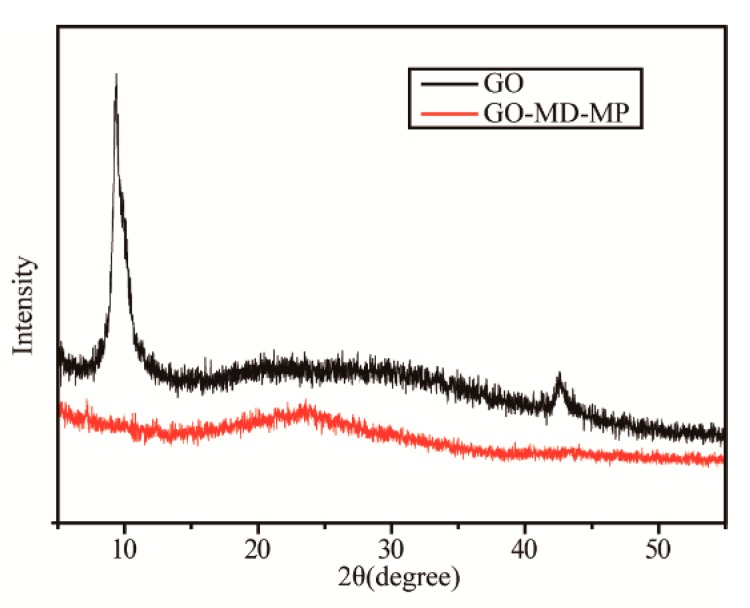
XRD patterns of GO and GO-MD-MP.

The XRD patterns of GO and GO-MD-MP are represented in Figure 6. The characteristic diffraction peak at 2θ = 9.5° was attributed to GO, corresponding to the (002) reflection of GO, indicating an interlayer distance of 0.93 nm. However, after the chemical modification of GO with PDPG, GO-MD-MP showed no visible diffraction peak in its XRD patterns, indicating that the GO-MD-MP sheet was separated and dispersed randomly, which may contribute to improving the dispersion of GO-MD-MP in epoxy resin.

**Figure 7:**
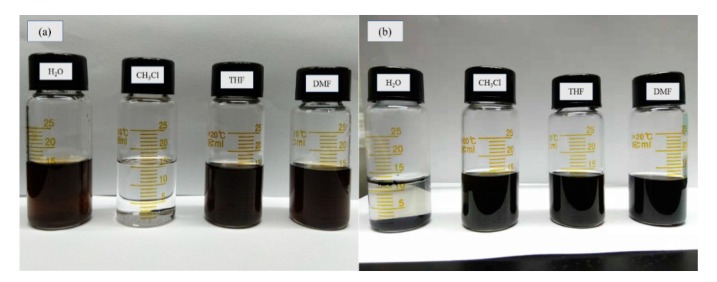
GO (**a**) and GO-MD-MP (**b**) in different solvents.

The dispersibility of modified graphene was a significant factor in the preparation of graphene-based composites. The enhancement of the nonflammability of graphene in composites also depended on the dispersion of graphene in the matrix. Figure 7 shows the dispersion of GO and GO-MD-MP in different solvents including water, chloroform, THF, and DMF. It shows that GO dispersed well and formed brown homogeneous suspensions in water, THF, and DMF. However, GO settled to the bottom when it was added to chloroform. After GO was modified with PDPG, the dispersions remained homogeneous in chloroform, THF, and DMF due to the good solubility of PDPG in chloroform, THF, and DMF. On the contrary, the hydrophobic group in PDPG on the graphene surface resulted in GO-MD-MP, in water, settling to the bottom of the serum bottle. The results indicate that functionalized graphene with PDPG can improve the solubility and compatibility of GO in organic solvents. It is worth mentioning that the excellent dispersion of GO-MD-MP in chloroform, THF, and DMF is helpful to prepare EP/GO-MD-MP composites, because chloroform, THF, and DMF are also good solvents for epoxy resin.

**Figure 8:**
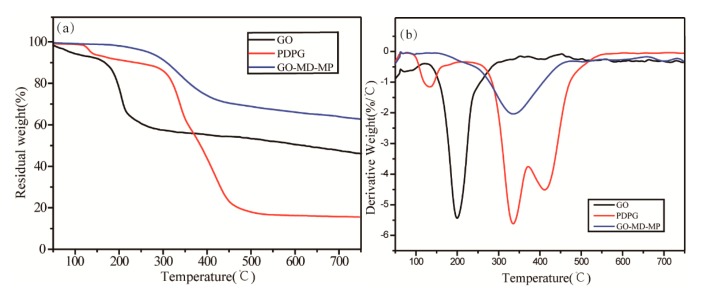
The thermogravimetric analysis (TGA) (**a**) and derivative weight (DTG) (**b**) curves of GO, PDPG, and GO-MD-MP.

The thermal degradation curves of GO, PDPG, and GO-MD-MP were investigated using TGA under nitrogen atmospheres. As shown in Figure 8, the mass loss of GO started below 100 °C, and the main weight loss of GO was around 200 °C, which were attributed to the loss of the carboxyl groups, hydroxyl epoxy and hydroxyl, on GO and indicated the insufficient thermal stability of GO. In addition, the char yield ratio of GO was just 43.02% at 700 °C. For PDPG, a mass loss around 120 °C appeared and was attributed to GMA degradation due to the catalytic action of the phosphorus flame retardant. The main mass loss was between 300 and 450 °C, which was due to the degradation of POSS and DOPO. The char yield ratio of PDPG was about 15.70%. When GO was grafted with PDPG, its main weight loss temperature was about 330 °C due to the high bond energy of Si–O and Si-C in the POSS skeleton structure, reflecting that the thermal properties of GO-MD-MP had been significantly improved. On the other hand, the char yield ratio of GO-MD-MP was enhanced to 64.69%, compared to pristine GO, which suggests that when GO is grafted with PDPG its thermal properties are improved.

**Figure 9:**
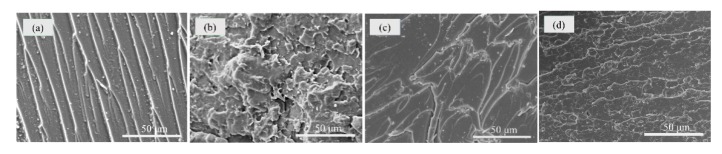
The SEM images of the fractured surface of pure epoxy resin (EP) (**a**), EP-4% PDPG (**b**), EP-2% GO-MD-MP (**c**), and EP-4% GO-MD-MP (**d**) composites.

The dispersion and interface strength of the graphene-based nanofillers in the polymer matrix are key factors for the properties of functionalized graphene/polymer nanocomposites. The SEM images of the fractured surface of the graphene/polymer nanocomposites is usually used to evaluate the dispersion of functionalized graphene [35]. Figure 9 shows the fractured surface of different cured EP composites. It is commonly known that pure epoxy resin is a brittle material and this was found to be the case in our system, as shown in Figure 9a. When PDPG was added, a lot of curved lines were observed on the fractured surface of the EP composites, which enhanced their toughness. However, some obvious protrusions were on the surface, which resulted in a poor interface strength between PDPG and the EP matrix, which may generate poor mechanical properties. On the contrary, after grafting PDPG to GO, the surface of the EP composites was rough without obvious protrusions, indicating that the interface strength of GO-MD-MP in the EP matrix was enhanced significantly. This may be explained by the fact that PDPG can weaken the intermolecular forces between different nanosheets of graphene. In addition, the covalent bonds connecting GO and PDPG may prevent POSS from aggregating, resulting in a better compatibility between GO-MD-MP and the EP matrix.

### 3.2. Thermal Properties and Flame Retardance of GO-MD-MP/EP Composites

**Figure 10:**
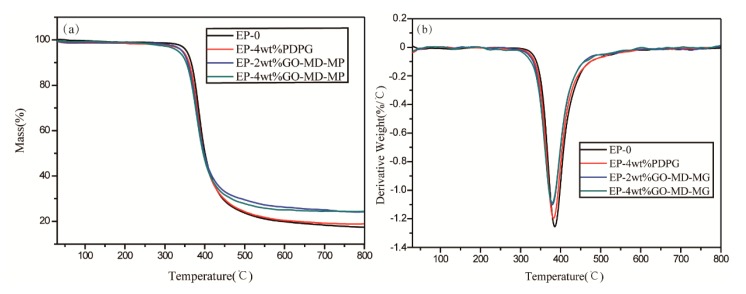
The TGA (**a**) and DTG (**b**) curves of the different cured EP composites.

The TGA and DTG curves of the different cured EP composites under nitrogen atmospheres are shown in Figure 10. In Figure 10a, all cured EP composites had a mass loss process that occurred between 300 and 500 °C, which was the decomposition of the epoxy resin curing network. The initial decomposition temperature (*T*_d_) can be adapted to the ambient temperature of epoxy resin. As is shown in Figure 10b, the maximum weight loss rate of GO-MD-MP/EP composites was evidently lower than the pure EP and PDPG/EP composites, indicating that the addition of GO-MD-MP can delay the thermal degradation process of epoxy resin. In addition, the residue chars at 700 °C of the pure EP and EP-4% PDPG were at 17.40% and 18.52%, respectively. However, the residue chars of the EP-2 wt % GO-MD-MP and EP-4 wt % GO-MD-MP were 25.50% and 25.77%, which were higher than that of the pure EP and EP-4 wt % PDPG due to the excellent thermostability of GO-MD-MP.

**Figure 11:**
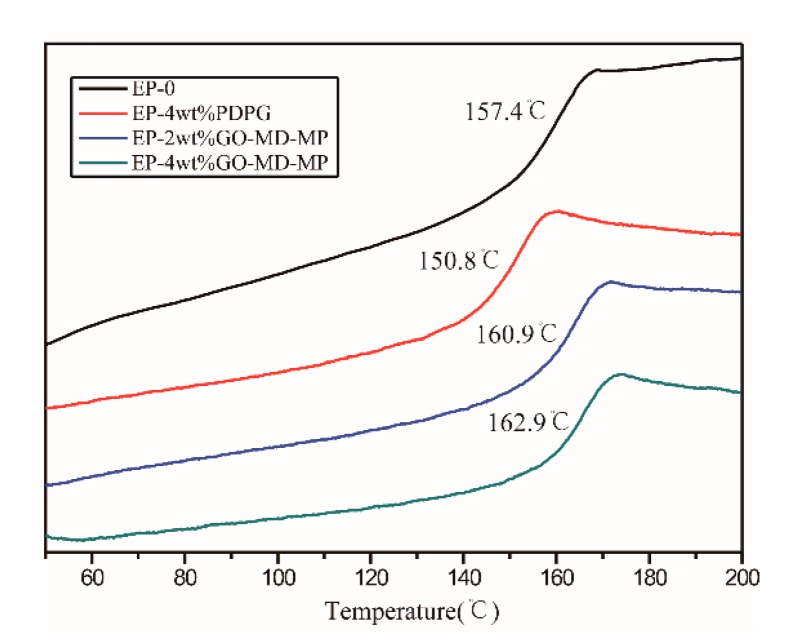
The DSC curves of pure EP and its different cured EP composites.

Glass transition temperature (*T*_g_) is a significant parameter to evaluate the performance and process properties of a polymer. The DSC curves of the pure EP and its different cured EP composites are shown in Figure 11. The *T*_g_ value of the pure EP was 157.4 °C. After adding PDPG into EP, the *T*_g_ values decreased, which may be attributed to the increasing free volume of the system caused by the large volume of POSS groups. However, when GO-MD-MP was added into EP, the *T*_g_ values increased slightly, which was because the effective dispersion of functionalized graphene achieved high aspect ratios in the EP composites, thus resulting in a higher cross-linking density.

The flame retardance of the resulting EP composites was tested by the determination of LOI values and the UL-94 test, and the results are presented in Table 2. As is shown in Table 2, the LOI value of pure EP was about 23.4%, indicating that it was very easy to burn. The 4 wt % addition of PDPG in the EP composites resulted in a slight improvement in flame retardancy with a LOI value of 27.9% and a UL-94 rating of just V-1. In contrast, the LOI values of the 4 wt % addition of GO-MD-MP in the EP composites was significantly improved to 31.1% and the UL-94 rating was V-0, revealing that the GO-MD-MP/EP exhibited a great flame retardancy and further indicating that GO-MD-MP played a significant role in the flame retardancy of the epoxy resin. It is worth noting that the same amount of PDPG (4 wt %) and GO-MD-MP (4 wt %) in the EP composites led to a huge difference of flame-retardant EP, and the flame retardancy of EP-4wt%GO-MD-MP was more remarkable than of EP-4 wt % PDPG. Furthermore, the content of the flame-retardant elements, P and Si, in GO-MD-MP is significantly lower than that in PDPG. It is indicated that graphene plays an important role for increasing flame retardancy, which is attributed to not only its excellent barrier properties caused by the unique platelet morphology of graphene, but also its tortuous properties resulting from the effective dispersion of GO-MD-MP in the EP composites [36].

**Figure 12:**
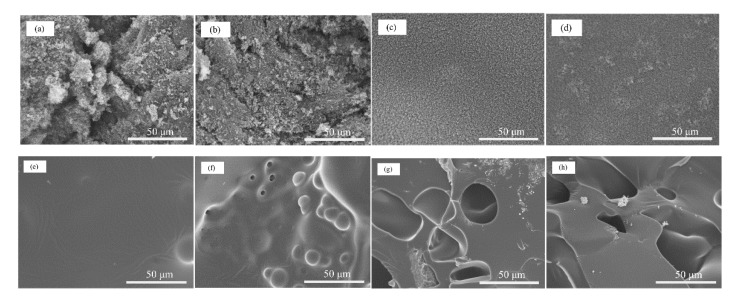
The SEM images of the outer (**a**–**d**) and the inner (**e–f**) residue chars of the pure EP (**a**,**e**), the EP-4% PDPG (**b**,**f**), the EP-2% GO-MD-MP (**c**,**g**), and the EP-4% GO-MD-MP (**d**,**h**) composites.

Figure 12 shows that the SEM images of the outer (a–d) and the inner (e–f) residue chars of the pure EP, the EP-4%PDPG, the EP-2%GO-MD-MP, and the EP-4%GO-MD-MP composites. It is shown that the residue char of the pure EP presented a loose outer layer and a smooth inner layer, which resulted from a lower amount of residue char, further indicating that the pure EP was fully degraded. The introduction of PDPG created a discontinuous outer char layer and a bubbly inner char, since many gaseous products escaped through the outer chars during combustion. In contrast, after adding GO-MD-MP, the outer residue chars of the cured EP composites were much more compact, which prevented the gaseous products escaping through the outer chars. Significant porosity was caused by the bubbles that were produced on the surface of the inner char, indicating that more gaseous products prevented the EP composites from burning. At the same time, these bubbles nucleated below the heated EP surface and could not destroy the outer residue chars. As a result, these residues formed a protective layer to protect the EP composites from burning.

**Figure 13:**
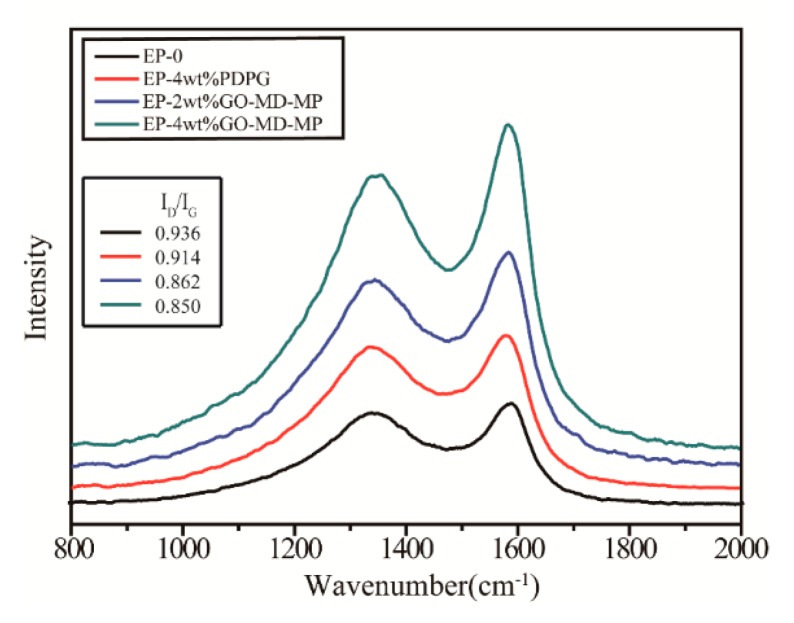
Raman spectra of different cured EP composites residue chars.

The graphitization degree of the residue char can be used to measure its thermal stability. Raman spectroscopy was conducted to measure the graphitization degree of the pure EP, the EP-4% PDPG, the EP-2% GO-MD-MP, and the EP-4% GO-MD-MP composite residue chars after treating them at 600 °C for 30 min in a muffle furnace. The corresponding Raman spectra are shown in Figure 13. The I_D_/I_G_ values of the four EP composites were 0.936, 0.914, 0.862, and 0.850, respectively. With the I_D_/I_G_ values decreasing in the EP composites, the graphitization degree of the residue char increased, which showed that the thermal stability of the residue chars improved gradually. These results indicated that both PDPG and GO-MD-MP can further improve the flame retardancy of EP composites. However, GO-MD-MP provided more compact residue chars with the same amount of addition.

### 3.3. Mechanical Properties

Finally, the mechanical properties of all the cured EP composites were measured via a three-point bending test. The flexural modulus and flexural strength of the EP composites were measured, and the results are shown in Table 3. The flexural modulus of the pure EP, EP-4% PDPG, EP-2% GO-MD-MP, and EP-4% GO-MD-MP was 2308.89, 2145.61, 2658.62, and 2738.46 MPa, respectively. At the same time, the flexural strength of the EP-4% GO-MD-MP composite increased by 15.88% relative to the pure EP, but the EP-4% PDPG decreased. The changes in the mechanical properties of different EP composites can be explained by the interface strength between the flame retardant and the EP matrix. As mentioned above, the poor interface strength of EP-4% PDPG led to the decrease of the flexural modulus. On the contrary, when GO-MD-MP was added into EP, the mechanical properties of the EP composites improved significantly due to the better interface strength between GO-MD-MP and the EP matrix.

## 4. Conclusions

In summary, a novel POSS-based copolymer modified graphene oxide hybrid flame retardant (GO-MD-MP) was successfully synthesized through grafting a POSS-based polymer (PDPG) to the GO backbone. The structure and morphology of GO-MD-MP was investigated by XPS, XRD, FT-IR, TEM, Raman spectroscopy, and TGA. The dispersion and exfoliation of modified graphene oxide was strikingly improved, resulting in a good dispersion of GO-MD-MP in the EP composites. For flame retardancy, the addition of 4 wt % GO-MD-MP to epoxy resin made it pass UL-94 with a V-0 rating. Meanwhile, the LOI value was greatly increased to 31.1%. The enhancement of the flame retardancy was mainly attributed to the excellent barrier function of the char and the tortuous properties that resulted from the effective dispersion of the graphene nanoplatelets, which can effectively prevent heat and oxygen from entering the inner epoxy matrix. As for the mechanical properties, the flexural modulus and flexural strength of the GO-MD-MP/EP composites were improved due to the excellent dispersion and interfacial interaction of modified GO in the EP matrix.

## Figures and Tables

**Table 1:** Composition and structure information of PDPG calculated by ^1^HNMR measurement.

Sample	Composition (mol %)			P (at%)	P/Si
	GMA	POSSMA	bisDOPOMA		
PDPG	16	18	66	1.8	0.92

**Table 2:** Limiting oxygen index (LOI) values and UL-94 data of the cured EP composites.

UL-94					
Samples	LOI (%)	Burning Grade	t1(s)	t2(s)	Dripping
**pure EP**	23.4	NR	>30s	-	Yes
**PDPG/EP-4%**	27.9	V-1	16.2	2.4	No
**GO-MD-MP/EP-2%**	29.4	V-1	19.6	3.8	No
**GO-MD-MP/EP-4%**	31.1	V-0	2.7	3.5	No

**Table 3:** Flexural modulus (MPa) and flexural strength (N·mm^−2^) of cured EP and its composites.

Samples	Flexural Modulus (MPa)	Flexural Strength (N·mm^−2^)	Percentage of Increased Flexural Strength (%)
**pure EP**	2308.89	142.451	0
**EP-4% PDPG**	2145.61	131.014	−8.03
**EP-2% GO-MD-MP**	2658.62	154.732	8.62
**EP-4% GO-MD-MP**	2738.46	165.067	15.88
